# Cost-minimization analysis of *GSTP1*c.313A>G genotyping for the prevention of cisplatin-induced nausea and vomiting: A Bayesian inference approach

**DOI:** 10.1371/journal.pone.0213929

**Published:** 2019-03-14

**Authors:** Ligia Traldi Macedo, Vinicius Eduardo Ferrari, Juliana Carron, Ericka Francislaine Dias Costa, Leisa Lopes-Aguiar, Gustavo Jacob Lourenço, Carmen Silvia Passos Lima

**Affiliations:** 1 Faculty of Medical Sciences, State University of Campinas (UNICAMP), Campinas, Brazil; 2 Centre for Economics and Administration (CEA), Pontifical Catholic University of Campinas (PUCC), Campinas, Brazil; Roswell Park Cancer Institute, UNITED STATES

## Abstract

**Background:**

Chemotherapy-induced nausea and vomiting are concerning adverse events resulting from cancer treatment, and current guidelines recommend the use of neurokinin-1-selective antagonists, such as fosaprepitant, in highly emetogenic schemes. However, the implementation of this strategy may be limited by the cost of treatment. *GSTP1* c.313A>G genotype was recently described as a predictor of vomiting related to high-dose cisplatin. We hypothesized that the inclusion of routine *GSTP1* c.313A>G screening may be promising in financial terms, in contrast to the wide-spread use of fosaprepitant.

**Methods:**

A cost-minimization analysis was planned to compare *GSTP1* c.313A>G genotyping versus overall fosaprepitant implementation for patients with head and neck cancer under chemoradiation therapy with high-dose cisplatin. A decision analytic tree was designed, and conditional probabilities were calculated under Markov chain Monte Carlo simulations using the Metropolis-Hastings algorithm. The observed data included patients under treatment without fosaprepitant, while priors were derived from published studies.

**Results:**

To introduce screening with real-time polymerase chain reaction, an initial investment of U$ 39,379.97 would be required, with an amortization cost of U$ 7,272.97 per year. The mean cost of standard therapy with fosaprepitant is U$ 243.24 per patient, and although the initial cost of routine genotyping is higher, there is a tendency of progressive minimization at a threshold of 155 patients (Credible interval–CI: 119 to 216), provided more than one sample is incorporated for simultaneous analysis. A resulting reduction of 35.83% (CI: 30.31 to 41.74%) in fosaprepitant expenditures is then expected with the implementation of *GSTP1* c.313A>G genotyping.

**Conclusion:**

*GSTP1* c.313A>G genotyping may reduce the use of preventive support for chemotherapy induced nausea and lower the overall cost of treatment. Despite the results of this simulation, randomized, interventional studies are required to control for known and unknown confounders as well as unexpected expenses.

## Introduction

Chemotherapy-induced nausea and vomiting (CINV) are dose-limiting adverse events that are reported in up to 80% of patients subjected to cancer treatment without additional support. [1] Severe CINV is linked to hospitalizations as well as quality of life impairment. [2,3] Hence, the prevention of CINV is of utmost importance in cancer care. Aprepitant and fosaprepitant (aprepitant prodrug) are neurokinin-1 (NK1)-selective antagonists and known to effectively reduce vomiting by blocking substance P brain-stem emetic activity. [4,5] These agents have been approved for over a decade by the Food and Drug Administration, following positive results from phase III trials demonstrating a reduction of approximately 20% in the risk of CINV in known emetogenic schemes. [6,7] Since then, most recommendation guidelines have included NK1 antagonists in addition to dexamethasone and 5-hydroxytryptamine (5-HT3) receptor antagonists for the prevention of CINV in highly emetogenic therapies, such as those including high-dose cisplatin (CDDP), [8] resulting in substantial reductions in CINV occurrence. [8,9]

On the other hand, the financial burden of cancer treatment remains a constant concern worldwide, [10–12] and the access to fosaprepitant and aprepitant for highly emetogenic chemotherapy schemes may be limited, particularly in developing countries, as a consequence of income restrictions. Possible strategies to address this major health concern could include the adoption of techniques related to precision medicine to better predict an individual’s response or tolerance to treatment, thereby selecting patients who would benefit from a certain therapy or support. In this case, pharmacogenomics could play a potential role in identifying benefit/risk groups. [13]

Recently, patients with head and neck cancer (HNC) under chemoradiation therapy with high-dose CDDP were prospectively evaluated in our institution, aiming to identify a possible correlation between single nucleotide polymorphisms (SNPs) involved in CDDP metabolism and the occurrence of CINV. [14] In this setting, the prophylaxis consisted of dexamethasone in combination with the 5-HT3 antagonist ondansetron, which is supported by the public health system in Brazil. Among the SNPs studied, the glutathione S-transferase P gene (*GSTP1*) c.313 (NM_000852.3, rs1695) was highlighted. In this prospective cohort, the AG or GG genotype conferred a 4.28 higher risk of CINV, with 46.7% of patients reporting grade 2 or greater events in contrast to 18.6% of patients with the AA genotype, thus demonstrating the potential value of this SNP as a predictor for CINV. Although this study is the first to report the association between *GSTP1* c.313 A>G and CINV secondary to cisplatin, similar results were suggested in a recent multifactorial risk model evaluation of a distinct chemotherapy scheme, considered to be highly emetogenic. [15] In the latter, 324 patients with breast cancer were submitted to FAC (combination of doxorubicin, 5-fluorouracil, and cyclophosphamide), in which the presence of the variant allele was related to an odds ratio (OR) of 2.20 higher risk of CINV, with borderline significance (95% Confidence interval 1.00–4.82, p = 0.049)

The *GSTP1* gene encodes the Pi1 protein involved in the inactivation of CDDP through its conjugation to glutathione, promoting cellular clearance. [16] Therefore, it is possible to hypothesize that functional polimorphisms of *GSTP1* may influence toxicity and treatment efficacy related to CDDP, as higher and longer intracellular exposure to active metabolites may occur. The possible relation of *GSTP1* c.313 A>G and overall toxicity to chemotherapeutical agents was already described in gastric and breast cancer regimens. [17,18] In a meta-analysis of 12 studies reporting the incidence of adverse events for patients with breast cancer, the presence of G allele for *GSTP1* c.313 A>G was associated with increased toxicity (OR 1.35, 95% Confidence interval 1.07 to 1.71, p = 0.011), including either hematological, gastrointestinal, neurological or non specified reports. [18] Because the protein encoded by the variant G confers reduced catalytic activity compared to that with the A allele, [16,19] the association of the AG or GG genotype with vomiting may be attributed to the accumulation of CDDP in epithelial enteroendocrine cells in the gastrointestinal tract. This results in serotonin secretion and stimulation of chemoreceptor trigger zones. [20,21]

The implementation of genotyping in daily practice is a developing field that could potentially bring benefit to patients regarding treatment decision making and reduction of overall costs. [13,22] Several studies have suggested the cost effectiveness of SNP assessments in the prevention of drug-related adverse events; however, no study related to CINV prediction has been performed to date. [23] Considering the favorable evidence for CINV screening using *GSTP1* and the costs of treatment, patient selection could be improved with the implementation of real-time-polymerase chain reaction (real-time PCR) for *GSTP1* in clinical practice. We thus performed this cost-minimization study in order to estimate the possible financial impact of *GSTP1* c.313A>G genotyping for predicting CINV risk assessment and selecting patients for fosaprepitant prescription.

## Materials and methods

### Decision analytic model

Chemoradiation with high-dose CDDP is considered the current standard therapy for HNC, either in neoadjuvant, adjuvant, or locally advanced disease settings. [24,25] The chemotherapy protocol considered for the study consists of intravenous CDDP at a dose of 100 mg per square meter of body surface area on days 1, 22, and 43 of radiotherapy, totaling three cycles. [26] Ideally, taking into account the high emetogenic risk of this regimen, fosaprepitant should be included as CINV prophylaxis as a premedication for all cycles. [8]

**Fig 1:**
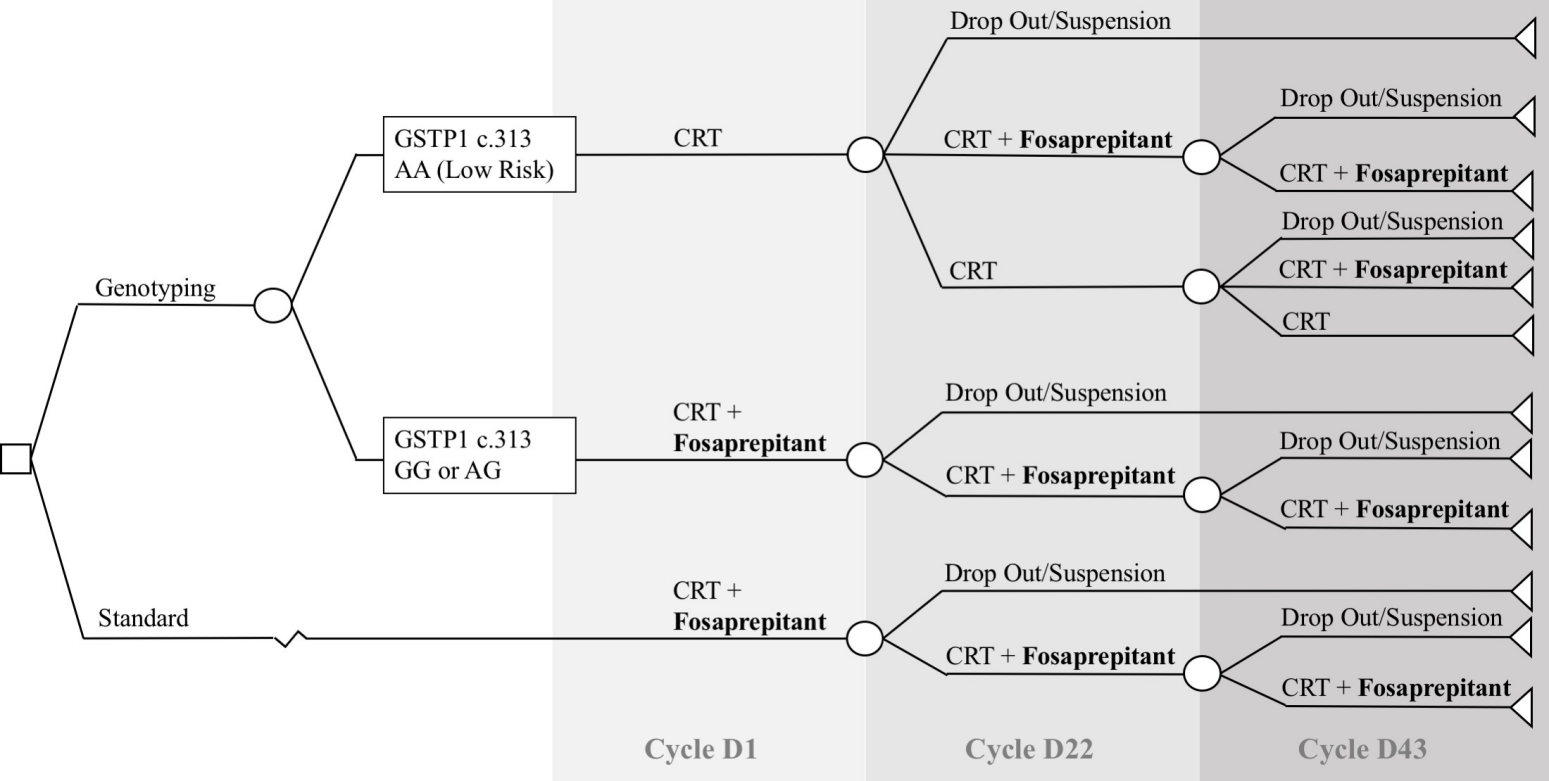
Decision analytic model. Visual representation of the decision analytic model, with the inclusion of *GSTP1* c.313 A>G genotyping for patient selection, prior to treatment initiation. In the genotyping branch, patients with AG or GG for *GSTP1* c.313 A>G (high risk) would receive fosaprepitant as a primary preventive measure. Patients with low risk (AA) genotype would receive fosaprepitant only in the presence of grades 3 or higher CINV, for subsequent cycles. The standard branch of the model represents the usual practice, without prior patient genotype assessment, including fosaprepitant since D1. *CRT*: *chemoradiation*.

With the hypothesis of including *GSTP1* c.313 A>G testing, a decision analytic model (Fig 1) was then applied to analyze the cost and transitional probabilities involved. In this visual representation, high-risk patients for vomiting (AG or GG) would be prescribed fosaprepitant in all cycles, while low-risk patients (AA) would receive the combination of dexamethasone and ondansetron, with the indication of fosaprepitant in subsequent cycles only in the presence of grade 3 or higher CINV, according to the National Cancer Institute (NCI)—Common Terminology Criteria for Adverse Events (CTCAE) version 4.03. The no genotyping (standard) branch represented the current standard of care, not involving genotype selection and including the recommended prophylaxis with fosaprepitant starting at treatment initiation (D1). For this conditional model, the following information was crucial:

- P1—the probability of a high-risk CINV genotype (or *GSTP1* c.313 A>G AG or GG);
- P2—the probability of grade 3 of higher nausea in patients not using fosaprepitant;
- The probability of continuing treatment in cycle 2 –D22 (P3) and cycle 3 –D43 (P4)

### Cost incorporation

The overall cost of treatment for both conditional settings was calculated following some preliminary suppositions. First, the analysis was based on the premise that the rate and dose of CDDP prescriptions, as well as those of additional support drugs (dexamethasone and ondansetron), would be similar between the two hypothetical branches (genotyping or standard) and could therefore be suppressed in the calculation. Furthermore, considering that only the consolidated percentage of CINV grades 3 or higher was described in the observed data and previously published evaluations, a constant rate of toxicity was then assumed for each prescription phase, with a consequent overestimation of this risk. The direct costs of medical agents, real-time PCR materials, and manpower are detailed in Table 1. The quotations for the chemotherapy regimen and support drugs were obtained in local currency (BRL) by consulting the local department of medical supplies and subsequently converted to US Dollars with the median exchange rate of 1 US$ for 3.70 BRL (Focus-BC report for 17 August 2018, from the Central Bank of Brazil).

**Table 1:** Cost incorporation summary.

Recurrent Costs	Overall Cost (US$)
***Treatment (price per unit)***	
Fosaprepitant 150 mg	$81.08
Dexamethasone 2,5 mL (4mg/mL)	$0.12
Ondansetron 8mg/4ml	$0.15
Cisplatin 1mg/ml 50 ml	$4.86
***Genotyping (with real-time PCR)***	
DNA extraction—reagents	$1.46
Real-time PCR—reagents	$8.56
Real-time PCR–materials	$1.07
***Manpower***	
Test specific and tax (per month) Test specific and tax (per hour)	$1,585.65 $9.91
**Investment and Amortization**	
StepOne Plus	$36,364.86
Annual amortization cost (20%)	$7,272.97

Summary of direct costs from recurrent (medical agents, PCR materials, and manpower) and investment expenditures in US Dollars.

For the costs of laboratory analysis, the information was gathered by the Cancer Genetics Laboratory (Laboratorio de Genética do Cancer—LAGECA) staff from our institution (State University of Campinas–UNICAMP), in US Dollars. For every real-time PCR procedure, four controls (one negative and three positive) were added. Potential losses of reagents and material were also incorporated, considering and addition of 10% from the original cost, for both DNA extraction and PCR. We carefully evaluated reagents’ durability and expiration dates for the estimation of quantities. Further details regarding the DNA extraction and real-time PCR acquisition are summarized in S1 and S2 Tables, respectively. Manpower was calculated under the assumption that the *GSTP1* c.313 A>G test would not be an exclusive task (S3 Table). Hence, the time for DNA extraction, real-time PCR, and data collection were calculated, and the cost of manpower was estimated according to the time consumed for laboratory analysis.

Additionally, aiming to better simulate clinical practice, we performed overall cost calculations considering either a single test, or simultaneous evaluations. The real-time PCR machine of choice is capable of performing 96 tests per turn, while it is possible to extract DNA from 12 samples simultaneously. In our service, receiving an average of 300 patients with this treatment indication per year, we considered seven days (six patients weekly) as the maximum time from the patient sample collection upon admission to the final result and decision making. We then performed simulations of up to six samples at once, and evaluated their respective cost reductions.

The initial investment included the necessary machinery for the real-time PCR *GSTP1* c.313 A>G testing (StepOnePlus Real-Time PCR System and Software, Thermo Fisher Scientific Inc.), assuming an annual amortization rate of 20%. [27] The overall value of the equipment was incorporated into the amortization, despite the strong possibility of other unrelated real-time PCR tests being performed with the same machine. Therefore, it is plausible to assume that the annual amortization cost would be lower than estimated in this analysis. However, we decided to preferably overestimate the expenditures taking into account the intangibility of additional procedures and savings.

### Statistical analysis

The cost-minimization analysis was performed by comparing the incremental cost for the inclusion of real-time PCR genotyping to the implementation of the standard CINV prophylaxis with fosaprepitant. In accordance with the Bayesian Bernoulli model, the posterior distribution of probabilities is computed from previously published sample sets (priors) and from binomial likelihood functions that describe the distribution of the selected data. In this case, the observed data consist of a prospective non-interventional study performed in the same institution, where 88 patients were submitted to high-dose CDDP and radiotherapy without the use of fosaprepitant. [14] For the prior risk of the *GSTP1* c.313 AG or GG genotype, we considered an additional prospective case-control study from a similar sample population of 229 patients. [28] The probability of grade 3 or 4 CINV was collected from a current meta-analysis (three-weekly CDDP arm), [29] while the chance of chemotherapy dropouts and suspensions was obtained from the intervention arm (N = 179) of a randomized clinical trial assessing high-dose CDDP and radiotherapy. [30] Only aggregated or de-identified data were used for this analysis, thus maintaining the confidentiality of the subjects involved in the included studies.

Following the probabilistic model assembly, Markov chain Monte Carlo (MCMC) simulations with 12,500 iterations using the Metropolis Hastings algorithm (MHA) were performed, with a burn-in phase of 2,500. [31] The MHA sampled repeatedly several random values extracted from the Bayesian Bernoulli model to estimate parameters–such as means, medians, and credible interval (CI)–for the posterior probabilities. The resulting means were then applied according to each corresponding hypothetical branch to calculate the total amount of fosaprepitant doses administered and the final respective costs (S1 Appendix). Trace plots, histograms, efficiency levels, and autocorrelation graphs were analyzed to validate the quality of the simulations. All calculations were performed using Stata/IC 15 (StataCorp LLC 2017. College Station, TX).

## Results

### Bayesian inferential calculations

The consolidated results from the MCMC simulations for each posterior probability are detailed in Table 2. The frequency of the *GSTP1* c.313 AG or GG genotype (high risk for CINV) was estimated to be 58.7% (CI from 53.8% to 63.4%), meaning that in the hypothetical genotyping branch, 41.3% of patients under chemoradiation therapy could be initially allocated to receive treatment without fosaprepitant. Regarding the chance of receiving subsequent chemotherapy cycles, 94.7% (CI from 91.7% to 97%) of patients were expected to continue treatment in D22 and 74.8% (CI from 69.2% to 80%) in D43. The rate of grades 3 or greater nausea during the entire treatment period for the simulation was 13.9% (CI from 10% to 18.1%). Consequently, it was calculated that the same percentage of patients would receive fosaprepitant in the following cycles, if continuing chemotherapy.

**Table 2:** Markov Chain Monte Carlo simulations.

	Observed Data (Binomial)		Prior (Beta)		Simulation Results		
Probabilities	Events	Total	Events	No Events	Mean	Median	Credible Interval
*GSTP1* High Risk (P1)	45	88	178	114	0.587	0.586	0.538–0.634
Grade 3/4 Nausea (P2)	4	43	33	191	0.139	0.138	0.100–0.181
Second Cycle Administration (P3)	86	88	160	12	0.947	0.948	0.917–0.970
Third Cycle Administration (P4)	64	86	120	40	0.748	0.748	0.692–0.800

Markov chain Monte Carlo simulations performed by Metropolis-Hastings algorithm, describing binomial, beta distributions, as well as the resulting proportions for each simulation and their respective credible intervals.

Notes: MCMC iterations = 12,500; Burn-in = 2,500; MCMC sample size = 10,000

All resulting diagnostic trace plots, histograms, efficiency levels, and autocorrelation plots are included in S2 Appendix.

### Sample quantity simulation

With the aim of replicating various scenarios from clinical practice, where patients may be included for PCR simultaneously, costs were calculated according to sample quantity for one PCR procedure. The detailed cost descriptions and time consumed for each laboratory stage are summarized in Table 3. Once more than one sample is included for genotyping, costs related to materials, manpower and reagents diminish. This decline can be explained by an optimization of manpower working time, and the use of less material regarding negative and positive controls.

**Table 3:** Costs per test according to number of samples.

Test Stages	Category	Sample Quantity per Test					
		1	2	3	4	5	6
**DNA Extraction (3.93 hours)**	Manpower	$38.98	$19.49	$12.99	$9.74	$7.80	$6.50
	Reagents^a^	$1.46	$1.46	$1.46	$1.46	$1.46	$1.46
**Real Time PCR** **(2.33 hours)**	Manpower	$23.12	$11.56	$7.71	$5.78	$4.62	$3.85
	Material^a^	$1.07	$0.64	$0.50	$0.43	$0.39	$0.36
	Reagents^a^	$8.56	$5.13	$3.99	$3.42	$3.08	$2.85
**Data Reporting** **(0.33 hours)**	Manpower	$3.30	$1.65	$1.10	$0.83	$0.66	$0.55
**Total cost per test**		$76.50	$39.94	$27.76	$21.67	$18.01	$15.57

Detailed costs regarding DNA extraction, real time PCR and data reporting considering samples per test. The time expected for each stage is also described.

^a^ Material and reagents costs were calculated with an addition of 10% expected losses and four controls.

**Fig 2:**
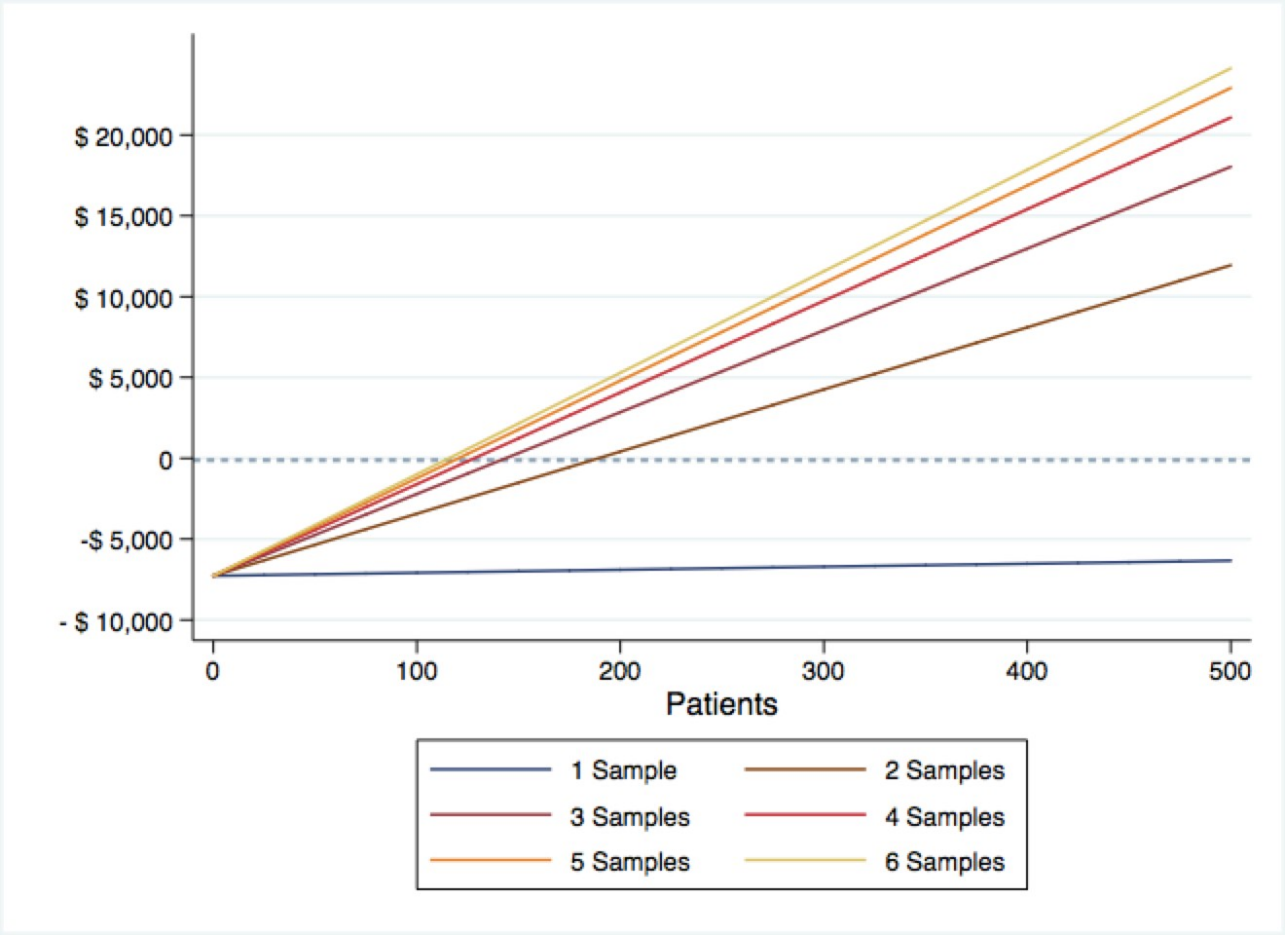
Cost reduction according to sample quantity. Cost reduction (overall cost from standard approach versus genotyping selection) calculations taking into account the number of samples per round.

Following the overall cost calculation, we then estimated the cost reduction for each scenario, as illustrated in Fig 2. It is possible to observe that the procedure of PCR selection becomes cost-beneficial once a minimum of two samples are incorporated. The maximum saving is obtained with the possibility of performing real-time PCR in six samples per turn. The time consumed from the patient inclusion and sample collection until the analysis of results will be dependable on the institutional rate of patient admissions. In a service with an average of 300 patients admitted per year, seven days would be the expected duration for the results to be retrieved.

### Recurring and amortization costs

The mean probabilities presented were used to estimate the total amount of fosaprepitant doses administered for each chemotherapy cycle. This procedure allowed the calculation of the overall fosaprepitant cost for both branches (Tables A and B in S3 Appendix). For the implementation of real-time PCR *GSTP1* c.313 A>G screening, an initial investment plan of U$ 39,379.97 is expected, with an annual amortization cost of U$ 7,272.97. The direct costs per test, corresponding to manpower and reagents, totaled U$ 39.94 for a two-sample and U$ 15.57 for a six-sample analysis.

### Cost comparisons

**Fig 3:**
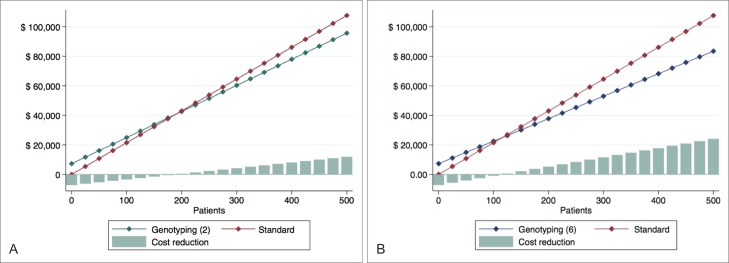
Overall cost for each hypothetical branch and cost reduction comparison. Overall cost from genotyping and standard approaches, once two (A) and six (B) samples are included for PCR.

The overall costs are presented in Fig 3. Although the cost of treatment with genotyping is initially higher than that of the standard branch, we observed a progressive reduction after a recovery from the annual amortization amount. Considering 300 patients as a proxy for the maximum number of new cases that can be treated per year in our institution and the performance of two samples, the overall cost of *GSTP1* c.313A>G testing would be US$ 60,314.65, in contrast to US$ 64,569.75 for the wide-spread prescription of fosaprepitant, resulting in a projected annual savings of US$ 4,255.10 (Fig 3A). On the other hand, annual savings progress as the incorporation of samples increases. If six samples are collected and analyzed every week, the overall cost would correspond to US$ 53,003.72, with a reduction of US$ 11,566.02 annually (Fig 3B).

**Fig 4:**
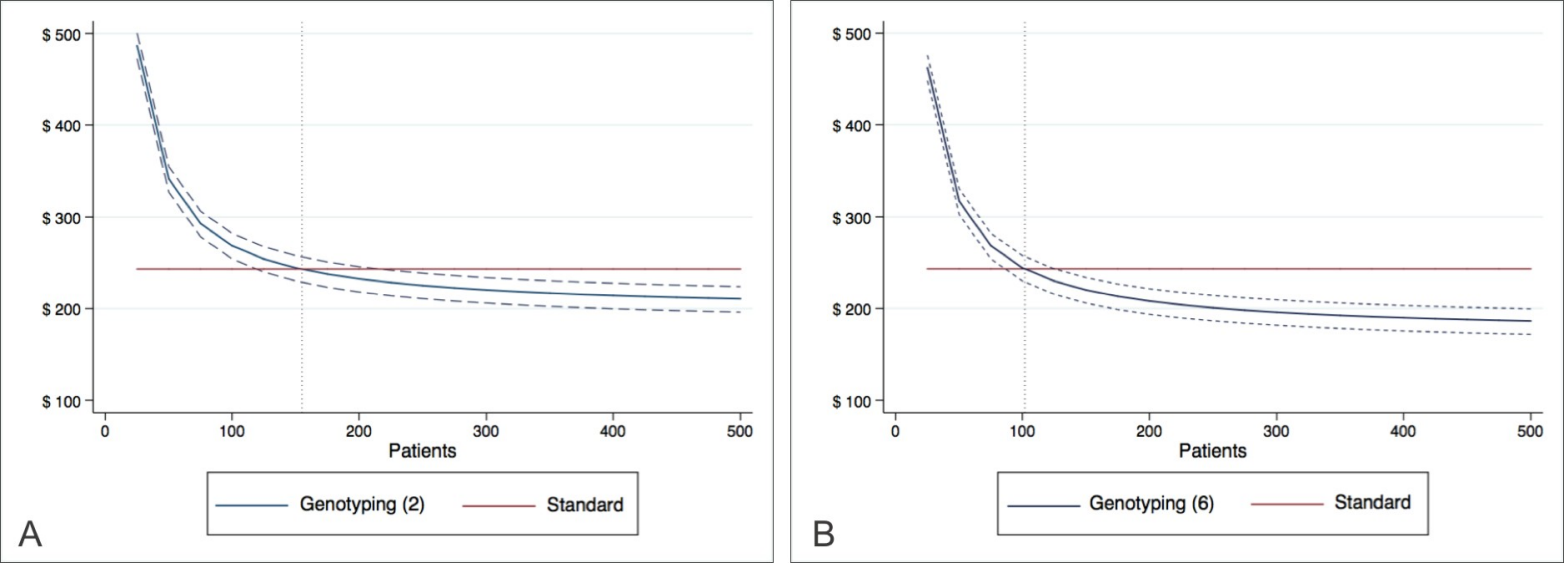
Overall cost per patient. Graphical representation of overall cost per patient, considering the complete treatment, in the setting of two-sample (A) and six-sample (B) analyzes, and their respective credible intervals. The vertical dotted reference line marks the patient threshold for cost-benefit.

The overall costs per patient for both treatment approaches are presented in Fig 4. By evaluating the simulation graph, it is possible to observe an escalating reduction with *GSTP1* c.313A>G testing. This finding can be explained by the drop in the amortization cost per test as patients are gradually included (see Table C in S3 Appendix, for more details). In the case of standard therapy, the cost of US$ 243.40 is constant, considering the use of fosaprepitant during all chemotherapy cycles.

The mean expense with fosaprepitant use decreases from US$ 243.40 to U$ 156.08 per patient (CI US$ 141.71 to US$ 169.52) with the real-time PCR test, resulting in a total reduction of 35.83% (CI 30.31 to 41.74%).

Moreover, the results indicate that given an annual amortization rate of 20%, 155 patients must be treated per year in order for the genotyping branch to become more advantageous (CI 119 to 216) for a two-sample analysis. Once this threshold is reached, there is a tendency of progressive cost minimization. An average difference of 9.45% (CI 3.92% to 15.36%) in the overall cost per patient is then expected with the implementation of *GSTP1* genotyping for 300 patients. In the setting of six-sample evaluations, the corresponding threshold is 102 (CI 85 to 126), resulting in an average difference of 19.46% (CI 13.94% to 25.37%).

## Discussion

There are more than 686,000 new cases of head and neck carcinomas every year worldwide. [32] Furthermore, the incidence is predicted to be higher in most developing countries. [33] The implementation of new technologies as well as supportive agents in cancer therapy has become a global challenge, [10,12] particularly for countries with low income rates, [11] despite current recommendations. Within this context, the development and adoption of safe selection criteria represents a potential strategy to address the financial burden of cancer care.

The *GSTP1* polymorphism was described as a promising predictor of CINV in a recent prospective evaluation, in which a subgroup of patients did not report concerning rates of nausea and vomiting even in the absence of fosaprepitant. [14] The present cost minimization study was then planned with the aim of more accurately evaluating and comparing the wide-spread implementation of fosaprepitant *versus* patient selection based on genotyping. The results of this simulation suggest a potential benefit in regards to limiting fosaprepitant prescription to higher CINV risk patients with the *GSTP1* AG or GG genotype, in services where the inclusion of simultaneous samples for PCR is possible without compromising the time for result analysis and treatment initiation.

There are, nonetheless, limitations to be considered in this study. One of the major restraints, already much discussed in the literature, relies on the principle that a SNP may not be the sole determinant of the metabolism of a specific drug; [13,34] other unknown SNPs could also play a key role in the response and toxicity, representing potential unassessed confounders. Consequently, several authors have suggested the use of a shared genetic database and whole genomic profiling to better characterize pharmacologic predictors. The resulting requirement for big data analysis has become one of the greatest challenges for current pharmacogenomics. [22,35] However, we believe that given the high frequency of *GSTP1* polymorphisms in the studied population, there is sufficient equipoise to support an interventional study with real-time PCR, in a randomized fashion, that can adjust for known and unknown confounders in a larger sample set.

There may be additional concerns related to the general applicability of these findings. Variability in drug material costs, toxicity rates, number of samples per test and even SNP frequency may alter the results of this simulation study. Structural heterogeneity, such as the presence or absence of a laboratorial facility, could further influence cost planning. Furthermore, unaccounted expenditures could play some role in the final cost analysis, although the price overestimation preferred by the authors for this cost minimization may act as a potential counterbalance.

In conclusion, *GSTP1* c.313A>G genotyping was demonstrated to be a promising predictor for CINV. This study shows a potential financial advantage to the application of real-time PCR for the selection of high-risk CINV patients, thereby suggesting the possible implementation of fosaprepitant for this subgroup. To confirm the assumptions in a cost-effectiveness study, the benefit of use of *GSTP1* genotyping should be demonstrated in a randomized, interventional study. In general, most SNPs remain restricted to observational data, hence limiting the assessment of detailed cost evaluations and their further implementation in routine practice. Regarding potential genomic predictors of response or toxicity and in the absence of safety concerns, more interventional studies on SNP testing can possibly increase the inclusion of pharmacogenomics in decision making, thus improving precision medicine in the clinic.

## Supporting information

S1 Appendix Equations for probabilities applying Bayes’ theorem. (PDF)

S2 Appendix Diagnostic trace plots, histograms, efficiency levels, and autocorrelation plots. (PDF)

S3 Appendix Overall and mean cost per patient simulations. (PDF)

S1 Table Acquisition costs and cost per sample for DNA extraction. Acquisition costs for DNA extraction reagents, in US Dollars. In this stage, no controls are required, and reagents are expected to be used until their expiration dates. Losses of 10% were incorporated into the original value.
(DOCX)

S2 Table Acquisition costs and costs per sample for real-time PCR reagents and materials. Acquisition costs for real-time PCR reagents and materials, in US Dollars. Reagents are expected to be used until their expiration dates. For every PCR stage, three positive and one negative controls were included.
(DOCX)

S3 Table Cost calculation for manpower. Detailed calculation for manpower cost per month in US Dollars.
(DOCX)
