# Supplementary material for: Cost-minimization analysis of GSTP1c.313A>G genotyping for the prevention of cisplatin-induced nausea and vomiting: A Bayesian inference approach
Source: PLoS One. 2019 Mar 14;14(3):e0213929. doi: 10.1371/journal.pone.0213929 (PMC6417645; doi:10.1371/journal.pone.0213929)
Supplement: S1 Appendix — (PDF) [file pone.0213929.s001.pdf]

Equations

Calculations of fosaprepitant administration in the PCR branch:

First Cycle:

$$N * P1$$

Second Cycle:

$$N * P1 * P3 + N * (1 - P1) * P2 * P3$$

Third Cycle:

$$N * P1 * P3 * P4 + N * (1 - P1) * (1 - P2) * P3 * P2 * P4$$

Calculations of fosaprepitant administration in the traditional branch:

First Cycle:

$$N$$

Second Cycle:

$$N * P3$$

Third Cycle:

$$N * P3 * P4$$

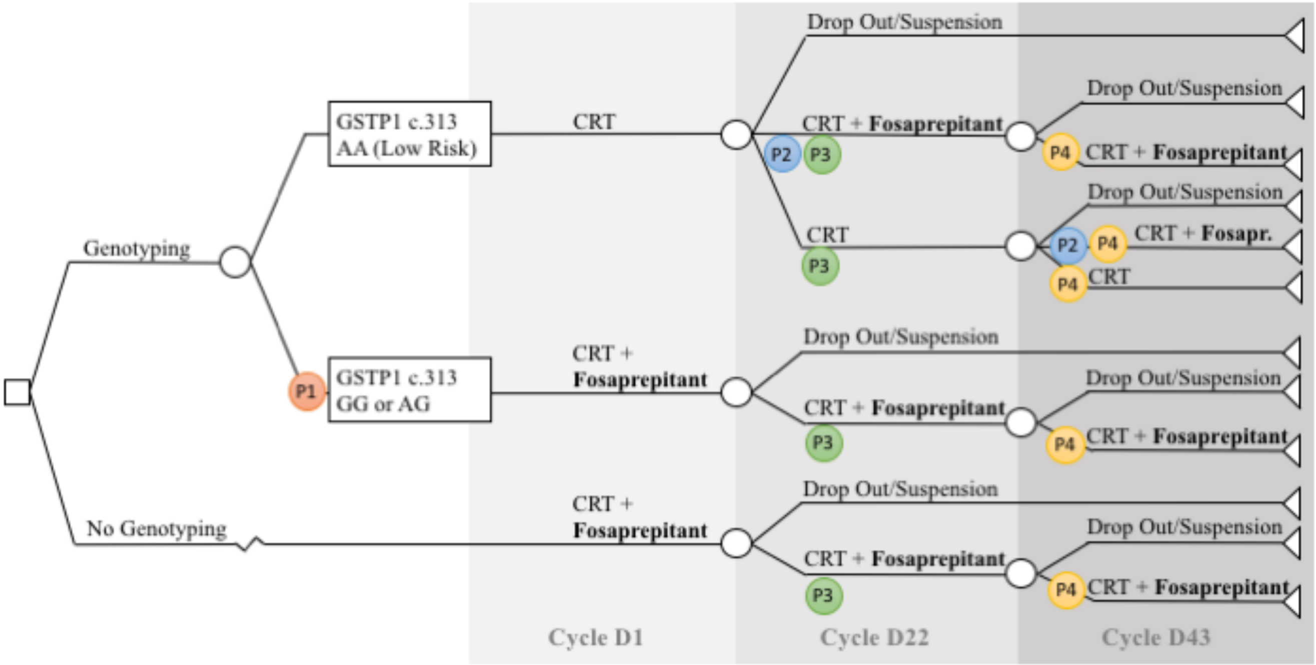

Legend:

P1: Probability of high risk GSTP1 genotype;

P2: Probability of Grades 3/4 Nausea after First and/or Second Cycles of chemotherapy;

P3: Probability of Concluding Second Cycle;

P4: Probability of Concluding Third Cycle.

N: Number of Patients
