# Supplementary material for: Cost-minimization analysis of GSTP1c.313A>G genotyping for the prevention of cisplatin-induced nausea and vomiting: A Bayesian inference approach
Source: PLoS One. 2019 Mar 14;14(3):e0213929. doi: 10.1371/journal.pone.0213929 (PMC6417645; doi:10.1371/journal.pone.0213929)

1. **Polymorphism Simulation (AG/GG) – Frequency**

Model summary

Likelihood:
  GSTP1_AG_GG ~ bernoulli({theta})

Prior:
  {theta} ~ beta(178,114)

Bayesian Bernoulli model   MCMC iterations =   12,500
Random-walk Metropolis-Hastings sampling   Burn-in   =   2,500
                                                                  MCMC sample size =   10,000

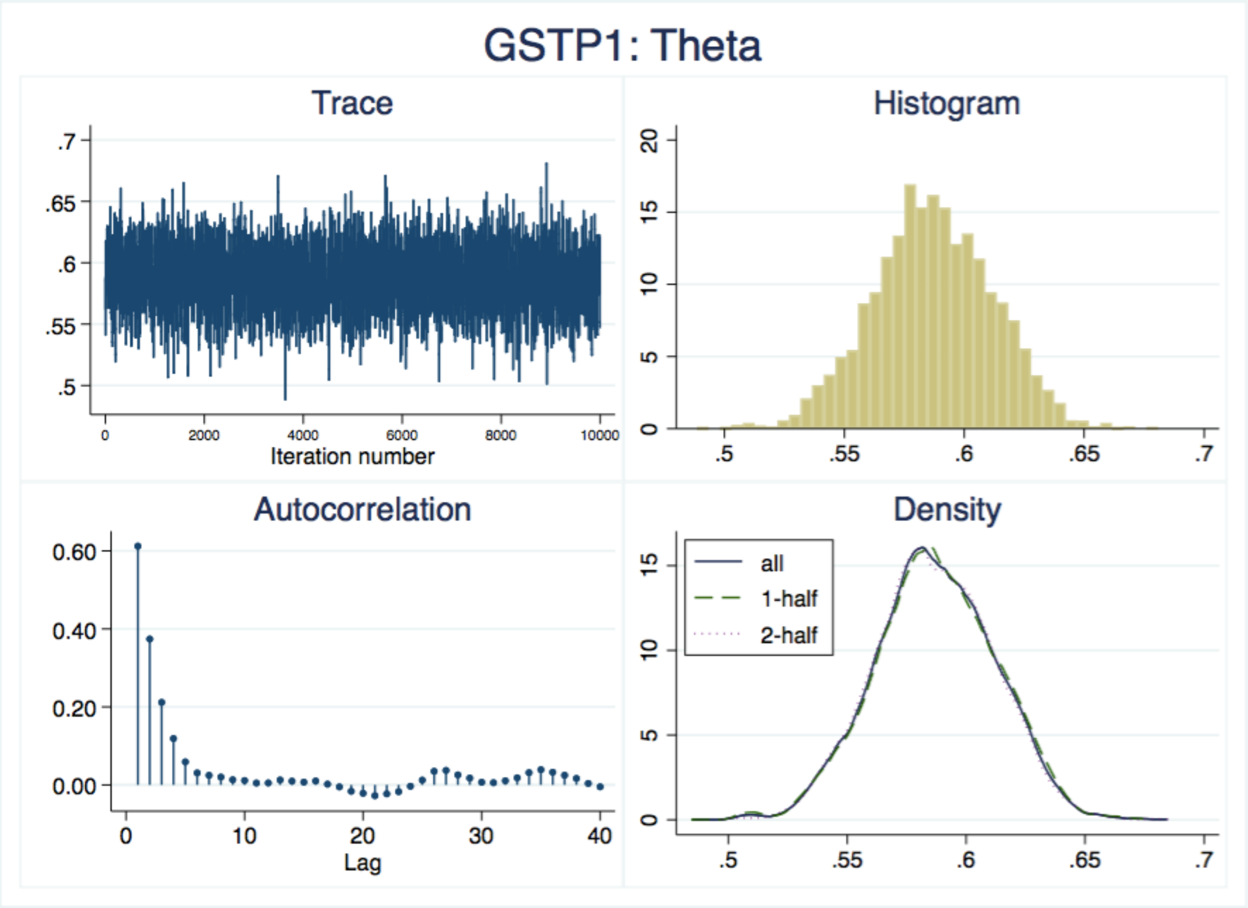

2. Grade ¾ Nausea Simulation

Model summary

Likelihood:
 NV ~ bernoulli({theta})

Prior:
 {theta} ~ beta(33,191)

Bayesian Bernoulli model MCMC iterations = 12,500
Random-walk Metropolis-Hastings sampling Burn-in = 2,500
MCMC sample size = 10,000

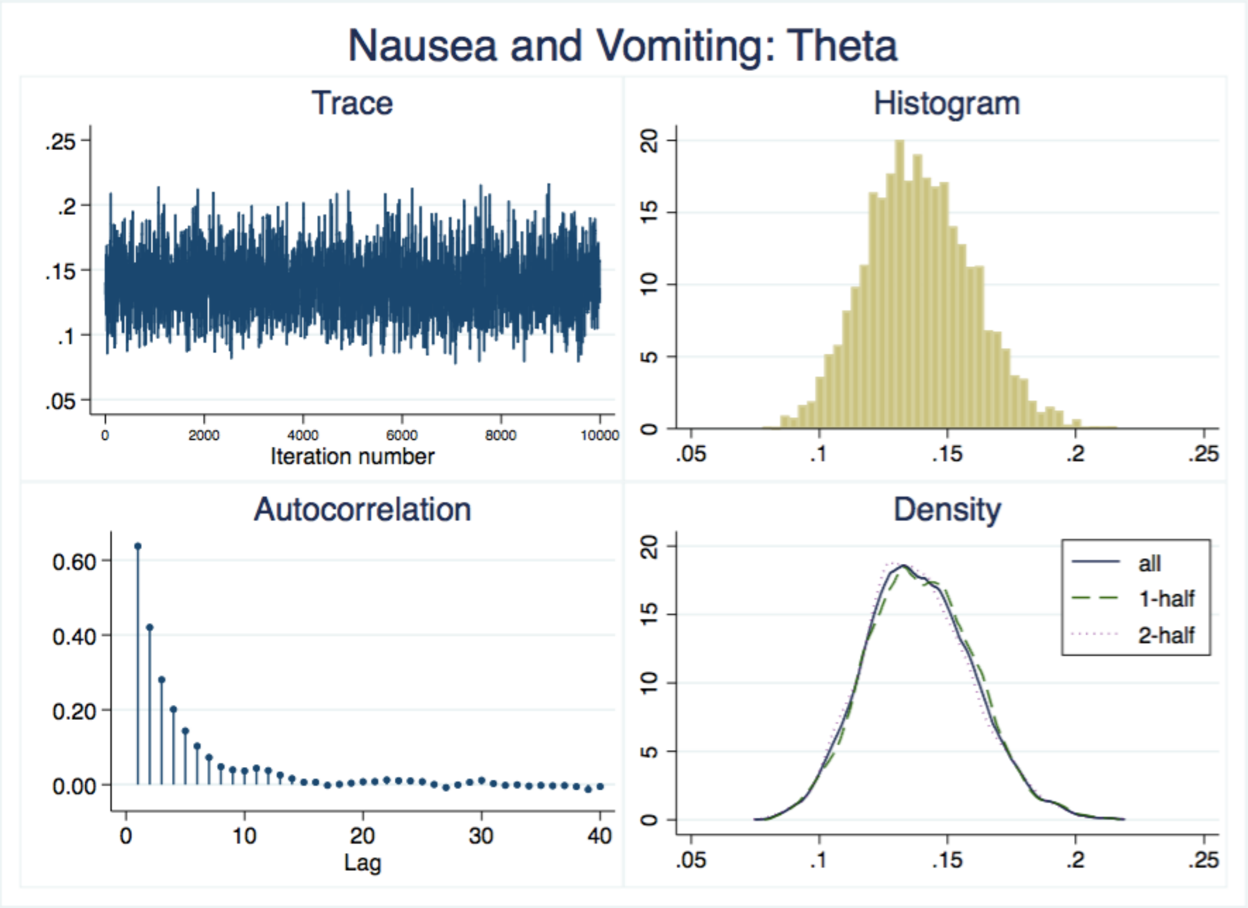

3. Continuing to Second Cycle Probability Simulation

Model summary

Likelihood:
  fase1 ~ bernoulli({theta})

Prior:
  {theta} ~ beta(160,12)

Bayesian Bernoulli model   MCMC iterations =   12,500
Random-walk Metropolis-Hastings sampling   Burn-in   =   2,500
                                                                        MCMC sample size =   10,000

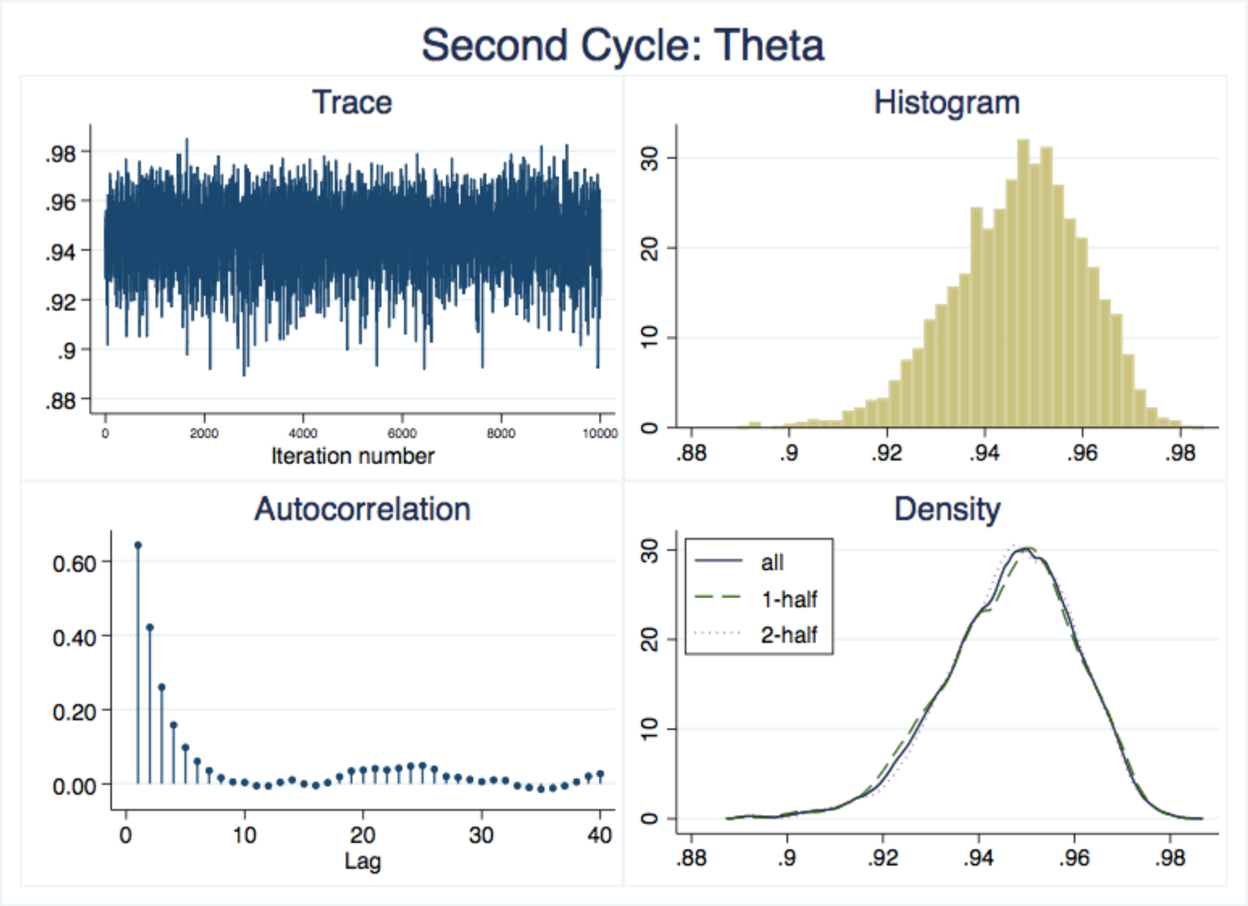

4. Continuing to Third Cycle Probability Simulation

Model summary

Likelihood:
  Continue3 ~ bernoulli({theta})

Prior:
  {theta} ~ beta(120,40)

Bayesian Bernoulli model MCMC iterations = 12,500
Random-walk Metropolis-Hastings sampling Burn-in = 2,500
MCMC sample size = 10,000

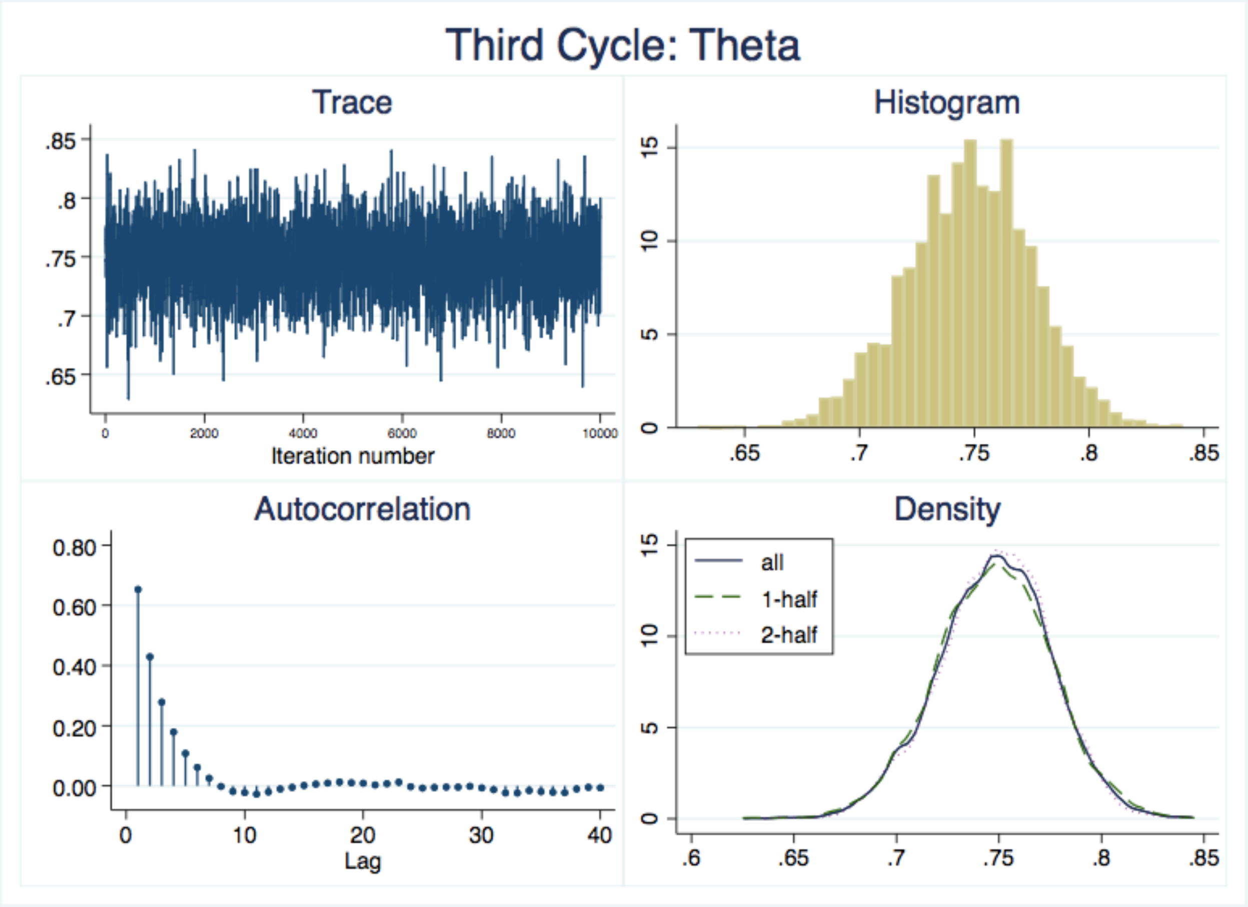

Supplement: S2 Appendix — (PDF) [file pone.0213929.s002.pdf]
