# Supplementary material for: Cost-minimization analysis of GSTP1c.313A>G genotyping for the prevention of cisplatin-induced nausea and vomiting: A Bayesian inference approach
Source: PLoS One. 2019 Mar 14;14(3):e0213929. doi: 10.1371/journal.pone.0213929 (PMC6417645; doi:10.1371/journal.pone.0213929)
Supplement: S3 Appendix — (PDF) [file pone.0213929.s003.pdf]

Supplementary file 3

A) Overall cost of treatment with genotyping in US Dollars

Patients	Fosaprepitant				2-Sample test		6-Sample test	
	Dose1	*Dose2*	*Dose3*	*Cost*	*Genotyping, amortization*	*Overall Cost*	*Genotyping, amortization*	*Overall Cost*
0	0	0	0	0	$7,272.97	$7,272.97	$7,272.97	$7,272.97
25	15	15	12	$3,421.57	$8,271.54	$11,693.11	$7,662.30	$11,083.87
50	29	30	25	$6,843.15	$9,270.11	$16,113.25	$8,051.62	$14,894.76
75	44	46	37	$10,264.72	$10,268.67	$20,533.39	$8,440.94	$18,705.66
100	59	61	49	$13,686.29	$11,267.24	$24,953.53	$8,830.26	$22,516.56
125	73	76	61	$17,107.86	$12,265.81	$29,373.67	$9,219.59	$26,327.45
150	88	91	74	$20,529.44	$13,264.37	$33,793.81	$9,608.91	$30,138.35
175	103	107	86	$23,951.01	$14,262.94	$38,213.95	$9,998.23	$33,949.24
200	117	122	98	$27,372.58	$15,261.51	$42,634.09	$10,387.56	$37,760.14
225	132	137	110	$30,794.16	$16,260.07	$47,054.23	$10,776.88	$41,571.04
250	147	152	123	$34,215.73	$17,258.64	$51,474.37	$11,166.20	$45,381.93
275	161	168	135	$37,637.30	$18,257.21	$55,894.51	$11,555.52	$49,192.83
300	176	183	147	$41,058.88	$19,255.77	$60,314.65	$11,944.85	$53,003.72
325	191	198	160	$44,480.45	$20,254.34	$64,734.79	$12,334.17	$56,814.62
350	205	213	172	$47,902.02	$21,252.91	$69,154.93	$12,723.49	$60,625.51
375	220	229	184	$51,323.59	$22,251.47	$73,575.07	$13,112.82	$64,436.41
400	235	244	196	$54,745.17	$23,250.04	$77,995.21	$13,502.14	$68,247.31
425	249	259	209	$58,166.74	$24,248.61	$82,415.35	$13,891.46	$72,058.20
450	264	274	221	$61,588.31	$25,247.17	$86,835.49	$14,280.78	$75,869.10
475	279	290	233	$65,009.89	$26,245.74	$91,255.63	$14,670.11	$79,679.99
500	293	305	246	$68,431.46	$27,244.31	$95,675.77	$15,059.43	$83,490.89

Formulas:

Dose 1: Patients*P1

Dose 2: Patients*P1*P3 + Patients*(1-P1)*P2*P3

Dose 3: Patients*P1*P3*P4 + Patients*(1-P1)*(1-P2)*P3*P2*P4

Cost Fosaprepitant: (Dose1 + Dose2+ Dose3)* $ 81.08

Genotyping and amortization (2-Sample test): Patients*39.94 + $ 7,272.97

Overall Cost (2-Sample test): Fosaprepitant + Genetic test and amortization (2-Sample test)

Genotyping and amortization (6-Sample test): Patients*15.75 + $ 7,272.97

Overall Cost (6-Sample test): Fosaprepitant + Genetic test and amortization (6-Sample test)

B) Overall cost of standard fosaprepitant prescription

Patients	Dose 1	Dose 2	Dose 3	Fosaprepitant Cost
0	0	0	0	0
25	25	24	18	$5,380.81
50	50	47	35	$10,761.62
75	75	71	53	$16,142.44
100	100	95	71	$21,523.25
125	125	118	88	$26,904.06
150	150	142	106	$32,284.87
175	175	166	124	$37,665.69
200	200	189	142	$43,046.50
225	225	213	159	$48,427.31
250	250	237	177	$53,808.12
275	275	260	195	$59,188.93
300	300	284	212	$64,569.75
325	325	308	230	$69,950.56
350	350	331	248	$75,331.37
375	375	355	265	$80,712.18
400	400	379	283	$86,093.00
425	425	402	301	$91,473.81
450	450	426	319	$96,854.62
475	475	450	336	$102,235.43
500	500	473	354	$107,616.24

C) Overall cost per patient

Patients	Fosaprepitant		2-Sample Analysis		6-Sample Analysis	
	Mean	*CI*	*Mean*	*CI*	*Mean*	*CI*
25	$156.08	$141.71 - 169.52	$486.94	$472.57 - 500.38	$462.57	$448.19 - 476.00
50	$156.08	$141.71 - 169.52	$341.48	$327.11 - 354.92	$317.11	$302.73 - 330.54
75	$156.08	$141.71 - 169.52	$293.00	$278.63 - 306.44	$268.63	$254.25 - 282.06
100	$156.08	$141.71 - 169.52	$268.75	$254.38 - 282.19	$244.38	$230.00 - 257.81
125	$156.08	$141.71 - 169.52	$254.21	$239.84 -267.65	$229.84	$215.46 - 243.27
150	$156.08	$141.71 - 169.52	$244.51	$230.14 - 257.95	$220.14	$205.76 - 233.57
175	$156.08	$141.71 - 169.52	$237.58	$223.21 - 251.02	$213.21	$198.83 - 226.64
200	$156.08	$141.71 - 169.52	$232.39	$218.02 - 245.83	$208.02	$193.64 - 221.45
225	$156.08	$141.71 - 169.52	$228.35	$213.98 - 241.79	$203.98	$189.60 - 217.41
250	$156.08	$141.71 - 169.52	$225.12	$210.74 - 238.55	$200.75	$186.37 - 214.18
275	$156.08	$141.71 - 169.52	$222.47	$208.10 - 235.91	$198.10	$183.72 - 211.53
300	$156.08	$141.71 - 169.52	$220.27	$205.90 - 233.71	$195.90	$181.52 - 209.33
325	$156.08	$141.71 - 169.52	$218.40	$204.03 - 231.84	$194.03	$179.65 - 207.46
350	$156.08	$141.71 - 169.52	$216.80	$202.43 -230.24	$192.43	$178.05 - 205.86
375	$156.08	$141.71 - 169.52	$215.42	$201.05 - 228.86	$191.05	$176.67 - 204.48
400	$156.08	$141.71 - 169.52	$214.21	$199.84 - 227.65	$189.84	$175.46 - 203.27
425	$156.08	$141.71 - 169.52	$213.14	$198.77 - 226.58	$188.77	$174.39 - 202.20
450	$156.08	$141.71 - 169.52	$212.19	$197.81 - 225.62	$187.82	$173.44 - 201.25
475	$156.08	$141.71 - 169.52	$211.34	$196.96 -224.77	$186.97	$172.59 - 200.40
500	$156.08	$141.71 - 169.52	$210.57	$196.20 - 224.01	$186.20	$171.82 - 199.63

*Considering only the patients that conclude the entire treatment (3 cycles of chemotherapy)

Overall cost per patient (2-Sample analysis), corresponding to: (1) the cost of Fosaprepitant; (2) the amortization cost per test, calculated by dividing the annual amortization cost by the initial number of patients; (3) the test costs concerning manpower and reagents ($ 39.94 per test).

Overall cost per patient (6-Sample analysis), corresponding to: (1) the cost of Fosaprepitant; (2) the amortization cost per test, calculated by dividing the annual amortization cost by the initial number of patients; (3) the test costs concerning manpower and reagents ($ 15.75 per test).
