# Supplementary material for: Cost-minimization analysis of GSTP1c.313A>G genotyping for the prevention of cisplatin-induced nausea and vomiting: A Bayesian inference approach
Source: PLoS One. 2019 Mar 14;14(3):e0213929. doi: 10.1371/journal.pone.0213929 (PMC6417645; doi:10.1371/journal.pone.0213929)
Supplement: S1 Table — Acquisition costs for DNA extraction reagents, in US Dollars. In this stage, no controls are required, and reagents are expected to be used until their expiration dates. Losses of 10% were incorporated into the original value. (DOCX) [file pone.0213929.s004.docx]

Acquisition costs and cost per sample for DNA extraction, in US Dollars

| **Reagents** | **Acquisition Cost** | **Quantity** | **Cost per Sample** | **Cost per Sample (10 % Loss)** |
| --- | --- | --- | --- | --- |
| Red Blood Cell Lysis (Buffer 1) | $ 0.69 / 1 L | 24 mL | $0.016 | $0.018 |
| Red Blood Cell Lysis (Buffer 2) | $1.34 / 200 mL | 1 mL | $0.005 | $0.006 |
| Genomic Digestion Buffer | $ 0.04 / 20 mL | 400 µL | $0.003 | $0.003 |
| Proteinase K | $36.16 / 250 mg | 20 µL | $0.143 | $0.159 |
| Lithium Chloride | $21.62 / 100mL | 200 µL | $1.081 | $1.201 |
| Ethanol (Absolute) | $27.03 / 1L | 1 mL | $0.027 | $0.030 |
| Ethanol 70% | $18.91 / 1L | 2 mL | $0.038 | $0.042 |
| TE Buffer | $0.02 / 1 mL | 30 µL | $0.003 | $0.003 |
| **Total** |  |  | **$1.316** | **$1.462** |
