# Supplementary material for: Cost-minimization analysis of GSTP1c.313A>G genotyping for the prevention of cisplatin-induced nausea and vomiting: A Bayesian inference approach
Source: PLoS One. 2019 Mar 14;14(3):e0213929. doi: 10.1371/journal.pone.0213929 (PMC6417645; doi:10.1371/journal.pone.0213929)
Supplement: S2 Table — Acquisition costs for real-time PCR reagents and materials, in US Dollars. Reagents are expected to be used until their expiration dates. For every PCR stage, three positive and one negative controls were included. (DOCX) [file pone.0213929.s005.docx]

| Real-time PCR (expected time per procedure: 140 minutes) | | | |
| --- | --- | --- | --- |
| Reagents | **Acquisition Cost** | **Cost per Sample*** | **Cost of Controls*** |
| Master Mix | $ 629.86 / 10 mL | $ 0.71 (10 µL) | $ 2.84 (40 µL) |
| Taqman Probe | $ 335.61 / 376 µL | $ 1.00 (1 µL) | $ 4.00 (4 µL) |
| MicroAmp Fast Tube, 0.1 mL | $ 127.01 / 125 units 8-Tube Strip | $ 0.14 (1 tube) | $ 0.56 (4 tubes) |
| MicroAmp Optical 8-Cap Strips | $ 158.93 / 300 units 8-Cap Strip | $ 0.07 (1 cap) | $ 0.29 (4 caps) |
|  | **Total** | **US$ 1.93** | **US$ 7.72** |

Acquisition costs and costs per sample for real-time PCR reagents and materials.

*considering 10% of potential losses
