# Supplementary material for: Cost-minimization analysis of GSTP1c.313A>G genotyping for the prevention of cisplatin-induced nausea and vomiting: A Bayesian inference approach
Source: PLoS One. 2019 Mar 14;14(3):e0213929. doi: 10.1371/journal.pone.0213929 (PMC6417645; doi:10.1371/journal.pone.0213929)
Supplement: S3 Table — Detailed calculation for manpower cost per month in US Dollars. (DOCX) [file pone.0213929.s006.docx]

| Manpower* |  |  |
| --- | --- | --- |
| Tax, Benefits, Salary, Time | **Value** |  |
| Salary | $ 869.20 |  |
| Meal Tickets | $ 256.76 |  |
| Thirteenth Salary | $ 72.43 |  |
| Holidays | $ 72.43 |  |
| 1/3 Holidays | $ 24.14 |  |
| Guarantee Fund (FGTS) | $ 83.06 |  |
| Tax INSS (20%) | $ 207.64 |  |
| Total work hours | 160 |  |
| Total | **U$ 1,585.65** |  |
| Total (per hour) | **US$ 9.91** |  |

Cost calculations for manpower in US Dollars.

*values per month
